# The Effect of Reciprocal Inhibition Techniques on Pain, Range of Motion, and Functional Activities in Patients With Upper Trapezitis

**DOI:** 10.7759/cureus.34487

**Published:** 2023-02-01

**Authors:** Swapna Jawade, Neha Chitale, Pratik Phansopkar

**Affiliations:** 1 Department of Musculoskeletal Physiotherapy, Ravi Nair Physiotherapy College, Datta Meghe Institute of Medical Sciences, Wardha, IND; 2 Dr. D. Y. Patil College of Physiotherapy, Dr. D. Y. Patil Vidyapeeth, Pune, IND

**Keywords:** met, upper back pain, physiotherapy intervention, musculoskeletal rehabilitation, trapezitis

## Abstract

Background and objective

Upper trapezius can cause neck pain, and restrict functional activities and cervical range of motion (ROM), and hence its management should be part of a global rehabilitation program. Owing to the heterogeneity of the existing trials, various techniques of manual physical therapy might be potent, though the scope of their efficacy is currently unspecified. The reciprocal inhibition technique of the muscle energy technique (MET) works on both agonist as well antagonist muscles for the reduction of pain and to improve overall functional activities. The aim of this study was to analyze the effect of the reciprocal inhibition technique of MET on pain, cervical ROM, and functional activities in patients with upper trapezitis.

Methods

An interventional cross-sectional study involving 30 patients with neck pain due to upper trapezitis was conducted. The outcome measures were as follows: numerical pain rating scale (NPRS) score for pain intensity, universal goniometer for cervical ROM, and neck disability index (NDI) score for functional activities. The reciprocal inhibition technique involved a five-second hold, five-second rest, followed by stretching with a 10-60-second hold, with five repetitions. Patients were treated for five sessions a week for two weeks.

Results

Paired t-test was used to compare the group's mean values before and after therapy. Our findings revealed that NPRS score, cervical ROM, and NDI score notably improved (p=0.001).

Conclusion

The reciprocal inhibition technique of MET in patients with upper trapezitis showed significant improvement in neck pain, cervical movement, and functional activities. Further studies with larger sample sizes are warranted to validate our findings.

## Introduction

The most extensive type of pain in non-traumatic musculoskeletal conditions is neck pain, with a frequency of about 75.7% [1,2]. It has become a major health problem in terms of personal health and the overall well-being of the public [3]. It affects 14.2-71% of individuals at some point in their lives [4]. Multiple pathologies are identified as causative factors for neck pain and one of the common factors is upper trapezitis. Trapezitis refers to the pain and spasm in the neck due to the inflammation of the trapezius muscle [5]. The most frequent musculoskeletal condition affecting people who work for extended periods in an uncomfortable neck position with repetitive movements is caused by pain and spasms in the trapezius muscles [6,7]. Upper trapezitis should be treated as part of a thorough physical therapy program since it has the potential to cause severe pain, restrict the range of motion (ROM), and impair functional tasks [8]. Upper trapezius muscles are frequently uncomfortable and tight in the neck region. Therefore, throughout the first phase of the treatment plan, emphasis should be placed on restoring the tight muscle to its normal length [9].

Several different therapy procedures can be used to treat upper trapezitis, but there is no consensus among the experts on the best course of action. The muscle energy technique (MET) is a type of manual physical therapy where the patient applies muscle contraction against the resistance provided by the therapist in a precisely controlled position and direction [10,11]. Indirect pressure on a joint always causes inhibition of one group of muscles in order to contract other groups of muscles, according to the reciprocal inhibition principle, which forms the basis of MET. Stretch receptors in the agonist muscle fibers and muscular spindle are responsible for this. This encourages joint mobility because of reciprocal inhibition [5,6].

The reciprocal inhibition approach has the benefit of being safe and effective in acutely or chronically tight and painful muscles that make controlled contraction of the affected muscles challenging [7]. In such cases, the therapeutic use of antagonists can be beneficial. Additionally, it helps when the agonist's muscles are frail [3]. Stretch receptors found in the agonist muscle fibers and muscular spindle are responsible for this as reciprocal inhibition promotes joint motion [5,6]. There is scarce literature on the effectiveness of the reciprocal inhibition approach in the treatment of upper trapezitis. Hence, the aim of this study was to investigate the effect of the reciprocal inhibition technique on neck pain, cervical ROM, and functional activities in the treatment of upper trapezitis.

## Materials and methods

Study design and setting

This study was conducted in the Department of Musculoskeletal Sciences, RNPC OPD & AVBR Hospital, Sawangi, Wardha. Cases of upper trapezitis seeking outpatient treatment in the Department of Musculoskeletal Sciences were selected according to the inclusion criteria. This was designed as an interventional cross-sectional study that was planned for one year with a sample size of 30.

Inclusion and exclusion criteria

Patients aged between 18 and 60 years who were willing to participate irrespective of gender with a pain intensity on NPRS between 4 to 8 and restricted cervical ROM were included. Patients with any particular causes of neck discomfort, such as tumors, intervertebral disc prolapse, pathology of the shoulder joint, neurological abnormalities affecting the upper limb, coagulation disorders, or trauma, and those who underwent surgery on the cervical spine in the previous 12 months, those with a history of cervical spine fractures or trauma, cervical radiculopathy or myelopathy symptoms, and vascular syndromes like VBI were excluded.

Methodology

Patients were given an information sheet in which the methodology of treatment was described in their local language. Only those patients willing to enroll were instructed to sign the informed written consent.

Before beginning the study, the institute's ethical committee gave its approval. The case history and physical conditions of the patients were thoroughly documented. Before starting treatment, numerous tests were done to rule out any other conditions. The outcome measures used in this study were cervical goniometry for ROM, the neck disability index (NDI) for functional activities, and the numerical pain rating scale (NPRS) for pain. The data were gathered at baseline, before the start of the intervention, and following the conclusion of each intervention session.

Intervention

Patients received care using the MET reciprocal inhibition method. Hot fomentation using a hot pack was given for 10 minutes. This was followed by five repetitions of reciprocal inhibition with 20% isometric contraction of the trapezius muscle for a five-second hold and five-second rest followed by the stretching of the upper trapezius muscle for a 10-60-second hold. The patients received the treatment for two weeks, five days a week [9]. The patients were placed in the supine position and the therapist stabilized the involved-side shoulder with one hand, while the other hand stabilized the involved-side ear/mastoid area. They were asked to side-bend the head and neck toward the opposite side, and then flex and rotate towards the same side. Then, the patients were positioned just short of the restriction barrier of the upper trapezius. They were directed to depress the affected side shoulder by using 20% of their maximum strength against the counter resistance provided by the therapist. They were then directed to hold the contraction isometrically for five seconds. The upper trapezius was extended for 10-60 seconds following isometric contraction [9,10,12,13].

Outcome measures

Pain Intensity

NPRS was utilized to determine the pain intensity. The numbers 0-10 were written on a 10-cm line, with 0 signifying no discomfort and 10 signifying the worst suffering. The maximum pain score was between 1 and 10 with 1 indicating the least pain and 10 signaling the most severe pain. The patients were requested to mark on the line according to their severity of pain [14].

Range of Motion and Functional Activities

The cervical movements were assessed using a universal goniometer for flexion, extension, lateral flexion, and rotation. NDI was used to evaluate functional activity limitation due to neck pain. In research and clinical contexts, NDI, which measures disability in individuals with neck discomfort, is crucial and is believed to have high reliability [15].

The data were recorded at the baseline and end of two weeks. The data were analyzed using IBM SPSS Statistics (IBM Corp., Armonk, NY). Proportion, mean, and standard deviation (SD) were analyzed using the chi-square test. Paired t-test was used to compare the group's mean values before and after therapy. A p-value of less than 0.05 was used to determine the significance level.

## Results

A total of 30 participants were included in the study, of which 18 were females and 12 were males. Two weeks of treatment protocol led to significant differences between pre- and post-pain scores. After two weeks, reciprocal inhibition resulted in a substantial decrease in pain (p=0.001). Following the reciprocal inhibition technique, the cervical ROM, including flexion, lateral flexion, extension, and rotation, considerably improved (p=0.001). Reciprocal inhibition led to statistically significant improvement in the NDI scores as all functional activities improved (p=0.001) (Table 1).

**Table 1:** Pre- and post-treatment values of pain, range of motion, and NDI Lat: lateral; Flex: flexion; NDI: neck disability index; SD: standard deviation

Outcome measures	Mean ± SD	P-value
Pre_pain	6.87 ± 0.937	0.001
Post_pain	2.03 ± 0.999	
Pre_flexion	41.03 ± 3.057 (degree)	0.001
Post_flexion	51.8 ± 3.231 (degree)	
Pre_extension	51.57 ± 3.52 (degree)	0.001
Post_extnsion	63.37 ± 3.168 (degree)	
Pre_Rt lat flex	28.08 ± 3.704 (degree)	0.001
Post_Rt lat flex	38.58 ± 3.26 (degree)	
Pre_Lf lat flex	27.03 ± 3.704 (degree)	<0.001
Post_Lf lat flex	39.41 ± 3.26 (degree)	
Pre_rotation	60.53 ± 4.946 (degree)	0.001
Post_rotation	73.5 ± 5.178 (degree)	
Pre_NDI	21.37 ± 6.387	0.001
Post_NDI	7.8 ± 4.429	

## Discussion

The current study was conducted to assess the effect of the reciprocal inhibition technique of MET on cervical pain, ROM, and functional activities limited due to neck pain. The reciprocal inhibition technique of MET results in the reduction of pain due to the stimulation of mechanoreceptors and proprioceptors by simultaneous isometric contraction and stretching of the muscles and this further makes consecutive stretch more tolerable and easier. The outcomes of this study in terms of pain reduction and improvement in cervical ROM and functional activities were comparable to those of earlier studies using MET's post-isometric relaxation approach, where the aforementioned aspects showed improvement in a wide range of neck-pain conditions. Based on previous studies on MET, the post-isometric relaxation technique has been most commonly used as the treatment technique of choice, and it has been highlighted in the literature that post-isometric relaxation of MET is notably efficacious in reducing pain and improving ROM and functional activities [4,5,6]. However, the efficacy of the reciprocal inhibition technique on upper trapezitis is yet to be determined and documented [16].

Phadke et al. examined the effects of the post-isometric relaxation technique on mechanical neck pain and found that MET significantly reduced pain and improved daily functioning [17]. Jalal et al. have stated that MET is beneficial in treating cervical discomfort and restricted ROM [18]. By increasing stretch tolerance and changing the viscoelastic characteristics of the soft tissue, MET alleviates pain and promotes flexibility [3]. A study by Gupta et al. compared the effects of post-isometric relaxation and isometric exercises on neck pain due to nonspecific causes and concluded that MET results in a significant improvement in pain intensity and functional activities [19]. Reciprocal inhibition led to a statistically significant improvement in NDI scores. NDI evaluates various aspects of cervical pain, including pain severity and overall routine functional activities. Reciprocal inhibition results in an improvement in NDI scores because of the reduction of pain and tightness in the trapezius muscle [20].

The management of upper trapezitis should be made part of a thorough rehabilitation program as it has the ability to cause pain, impede cervical ROM, and inhibit functional activities. Based on the outcomes of our study, clinicians may use reciprocal inhibition stretching as a component of their rehabilitation program, depending on the muscle that has to be treated. The reciprocal inhibition technique may also be used as a manual technique of choice in the treatment of upper trapezitis.

This study has a few limitations, primarily its small sample size and short duration. Further studies with longer duration of interventions and larger sample sizes are required to gain deeper insights into this topic.

## Conclusions

In patients with upper trapezitis, the reciprocal inhibition technique of MET demonstrated a significant improvement in terms of pain, cervical ROM, and functional activities throughout five sessions per week for two weeks. This study's findings endorse the utility of reciprocal inhibition-MET in managing patients with upper trapezitis.
